# Virtual Electric Dipole Field Applied to Autonomous Formation Flight Control of Unmanned Aerial Vehicles

**DOI:** 10.3390/s21134540

**Published:** 2021-07-01

**Authors:** Leszek Ambroziak, Maciej Ciężkowski

**Affiliations:** 1 Robotics and Mechatronics Department, Faculty of Mechanical Engineering, Bialystok University of Technology, Wiejska St. 45C, 15-351 Bialystok, Poland; 2 Automatic Control and Robotics Department, Faculty of Electrical Engineering, Bialystok University of Technology, Wiejska St. 45E, 15-351 Bialystok, Poland; m.ciezkowski@pb.edu.pl

**Keywords:** formation flight control, guidance, unmanned aerial vehicles, potential field, virtual electric dipole

## Abstract

The following paper presents a method for the use of a virtual electric dipole potential field to control a leader-follower formation of autonomous Unmanned Aerial Vehicles (UAVs). The proposed control algorithm uses a virtual electric dipole potential field to determine the desired heading for a UAV follower. This method’s greatest advantage is the ability to rapidly change the potential field function depending on the position of the independent leader. Another advantage is that it ensures formation flight safety regardless of the positions of the initial leader or follower. Moreover, it is also possible to generate additional potential fields which guarantee obstacle and vehicle collision avoidance. The considered control system can easily be adapted to vehicles with different dynamics without the need to retune heading control channel gains and parameters. The paper closely describes and presents in detail the synthesis of the control algorithm based on vector fields obtained using scalar virtual electric dipole potential fields. The proposed control system was tested and its operation was verified through simulations. Generated potential fields as well as leader-follower flight parameters have been presented and thoroughly discussed within the paper. The obtained research results validate the effectiveness of this formation flight control method as well as prove that the described algorithm improves flight formation organization and helps ensure collision-free conditions.

## 1. Introduction

Over the past decade, Unmanned Aerial Systems (UAS) have advanced greatly, mostly due to the dynamic progress in robotics, control engineering, telecommunication, and material sciences [1,2,3]. In general, the R&D community dealing with UAS has been facing several new issues, such as those connected with increasing autonomy levels [4,5,6,7], communication and data exchange systems [8,9,10], precise localization systems [11,12], propulsion and power systems [13,14], launchers and take off system [15,16,17], manned-unmanned teaming [18], interoperability [19,20,21], advanced control laws synthesis [22,23,24] and many others. However, within the last several years, a lot of attention has been focused on cooperative control, especially formation flight [25,26,27,28]. An effective and robust formation flight can strongly improve the operational potential of UAS [29,30]. Multiple UAVs that fly in formation and have the ability to work together can hold a prominent role in applications like military operations, map building, transport of heavy or bulky loads, search and rescue missions, aerial refueling, drag reduction, and energy savings or long-distance data retransmission. Efficient formation flying requires the development and testing of appropriate control laws. This is especially true in respect to small UAVs having low payload capacities which severely limit onboard sensor suites or flight computers and communication systems that they can carry. For this reason, control laws should be simple and provide reliable operation which, in turn, makes their design challenging. The problem of formation flight control has been well documented and widely considered in literature.

Formation flight control systems can be classified according to a number of criteria with the most important being the type of information they use and the way this information is exchanged. Examples include (i) systems based on a radio data exchange (most often concerning positions and velocities of objects within a group), (ii) those relying on video information that is obtained from cameras and computer vision systems [31,32], and (iii) systems based on specialized sensors - such as seekers [33]. Another manner of classifying formation control systems is through the structure of their control system and dependencies related to communication and data exchange. Three of the most commonly used structures are (i) leader-follower [34,35], (ii) virtual structure [36] and (iii) behavioral approach [37]. Flight formation control methods can also be categorized on the basis of the control system’s organizational arrangement (according to the place where decision making and control occurs), with examples including (i) centralized, (ii) decentralized, and (iii) hybrid. We can also divide the methods with regard to the type of control object and its kinematic properties. Methods can also be classified with respect to their control object, such as (i) nonholonomic [38,39] and (ii) holonomic, and its kinematic properties. There are also formations of homogeneous and heterogeneous objects [40].

In considering solely the technique used for formation flight control, it is possible to mention methods utilizing proportional-integral controllers [41,42,43], nonlinear [44,45] or with switching controllers [46]. Other types of controllers like optimal [47,48], adaptive [49,50], predictive [51] or robust [52] have also been analyzed and evaluated. In [53] and [54], for example, trajectory optimization methods for multiple agents of formation flight were presented and carefully discussed. An efficient and optimal path smoothing technique for UAV formation path planning that accounts for moving obstacles is proposed in [55] and for guiding a UAV to a desired position. However, artificial potential field derivation, their handling, and usage are carried out in various ways. Works [56,57,58,59], for example, utilized bifurcating potential fields to determine the desired airspeed and heading of a fixed-wing UAV and focused on keeping the aircraft on the desired trajectory defined as a constant radius circle or as a straight line. The authors of these works consider a decentralized control methodology without a leader. The [60], on the other hand, presents a virtual leader approach combined with an extended local potential field. This method was applied only with respect to small unmanned helicopters and is only suitable for holonomic mobile objects, greatly limiting its uses. The most frequently used methods of formation control represent the position or displacement-based approaches where objects measure their position or relative position within a global coordinate system. [61] proposes a formation control system that uses information concerning the line of sight angles but only with respect to nearby agents. Furthermore, it is important that the proposed algorithm does not require a communication link for data exchange between vehicles. A similar idea for controlling the formation of flying robots without relative position determination of each vehicle was proposed in [62] where the detective deviated pursuit rule regulates the distances between vehicles.

The present work also addresses the problem of UAV formation flight control. The algorithm proposed within the work uses a potential field to determine the desired heading for the follower UAV. A great advantage of this method is the ability to rapidly change this potential field function depending on the position of an independent leader. What is more, the potential field is generated in a manner that provides collision avoidance during formation flight. The system is decentralized and based on a leader-follower configuration and could be easily adapted to vehicles with different dynamics without having to retune heading control channel gains and parameters. One of the paper’s main goals was to conduct simulation tests of the proposed formation flight control system. The leader–follower simulation onboard control system was designed in a manner that corresponds to commercial autopilots and was carefully described in the paper. The synthesis of the potential field control algorithm is also closely described and presented in detail. The proposed control system was thoroughly tested and verified during simulations. Longitudinal and lateral positions of the UAVs (leader and follower) obtained during the tests were recorded and then presented on plots. Furthermore, waveforms of position errors were introduced to show control system quality. Generated vector fields, as well as the logged leader and follower flight parameters, were subjected to careful consideration. The Obtained results validate the effectiveness of the vector field-based formation flight control method and the developed control algorithm improves formation flight organization, provides a simple and computationally efficient method for controlling, and helps to ensure collision-free flight of multiple UAVs.

The remainder of the paper is organized in the following manner: Section 2 includes a thorough description of the research object, its mathematical model, and simulation system while the main definitions of the virtual electric dipole field and formation flight problem are formulated in Section 3. Section 4 presents the validation of the proposed navigation strategy conducted using computer simulations and illustrative examples. Comprehensive conclusions are presented in Section 5.

## 2. Research Object

### 2.1. The Bullit 60

**Figure 1:**
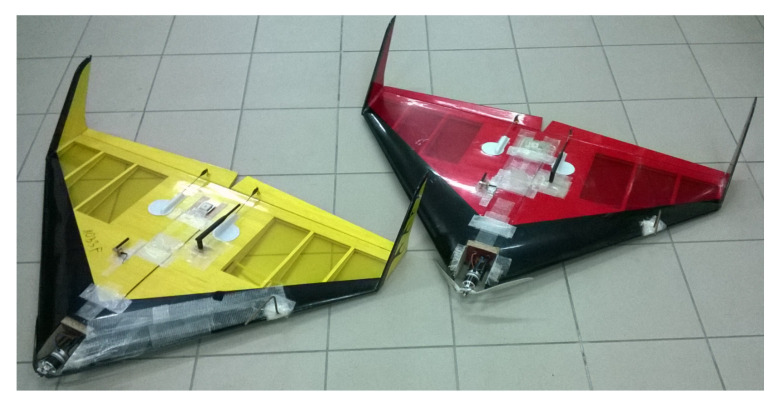
Bullit 60 MAV used in the studies.

The study utilized the Bullit 60, a micro air vehicle (MAV) [63] (Figure 1), which is a fixed-wing aircraft of the flying wing configuration. The control surfaces in a flying wing are commonly called elevons. The angular deflections of the right and left elevons are labeled as **$\delta_{e_{r}}$** and **$\delta_{e_{l}}$**, respectively. Using the following formulas, we can convert elevon singnals into a standard aileron–elevator (**$\delta_{a}$** and **$\delta_{e}$**) signals [64,65,66]:

$$\begin{array}{c} \delta_{e}=\delta_{e_{r}}+\delta_{e_{l}}\\ \delta_{a}=-\delta_{e_{r}}+\delta_{e_{l}} \end{array}.$$

Therefore, the mathematical model of the MAV presented in the next paragraph will be expressed in terms of standard aileron-elevator notation. The Bullit 60 is made of balsa wood covered with plastic skin and is powered by a front-mounted electric motor. Its propulsion system is composed of a two-blade, 14${}^{\prime \prime }$× 8${}^{\prime \prime }$ propeller that has an area (*S${}_{p}$*) of 0.0031 m${}^{2}$. The MAV’s wing is configured as a single-delta and has a symmetrical, bi-convex BELL 540 (modification of NACA0012) profile. The wing span (*b*) of the Bullit 60 is 1.095 m, whereas the wing area (*S*) is 49.7 dm${}^{2}$. The other important aerodynamic wing parameters are the mean chord (*c*) and the tip chord that are 0.660 m and 0.170 respectively. The total length (*l*) of the UAV is 0.770 m and its total ready-to-fly mass (*m*) is 1.8 kg. The vehicle’s maximum airspeed (*V*${}_{max}$) is 34 m/s and and its minimum velocity (*V*${}_{min}$) is 11 m/s.

### 2.2. Mathematical Model

To characterize the spatial motion of a UAV it is crucial to consider the action of forces and moments on the vehicle. To complete the mathematical model, it is also important to account for kinematic relations.

The considered UAV is symmetrical within the x–z plane. Consequently, the inertia matrix (please refer to Table 1) can be expressed as:

(1) $$\hat{I}=\left(\begin{array}{ccc} I_{x} & 0 & I_{xz}\\ 0 & I_{y} & 0\\ -I_{xz} & 0 & I_{z} \end{array}\right).$$

The six-degree-of-freedom, 12-state model with externally applied forces and moments due to gravity, aerodynamics, and propulsion system can be presented through Equations (2)–(5). Aerodynamic parameters (lateral and longitudinal) of the Bullit 60 were calculated using Advanced Aircraft Analysis (AAA) computer software developed by the DAR Corp. [67] and the Tornado VLM software [68] and were compared with wind tunnel experiments [69]. The Bullit equations of motion that have been applied in the present study are as follows:

(2) $$\left[\begin{array}{c} \dot{x}\\ \dot{y}\\ \dot{z} \end{array}\right]=Q_{1}\left[\begin{array}{c} u\\ v\\ w \end{array}\right],$$

(3) $$\left[\begin{array}{c} \dot{u}\\ \dot{v}\\ \dot{w} \end{array}\right]=\left[\begin{array}{c} rv-qw\\ pw-ru\\ qu-pv \end{array}\right]+Q_{2},$$

(4) $$\left[\begin{array}{c} \dot{\phi }\\ \dot{\theta }\\ \dot{\psi } \end{array}\right]=Q_{3}\left[\begin{array}{c} p\\ q\\ r \end{array}\right],$$

(5) $$\left[\begin{array}{c} \dot{p}\\ \dot{q}\\ \dot{r} \end{array}\right]=Q_{4}+Q_{5},$$

where:

$$Q_{1}=\left[\begin{array}{ccc} cos\theta cos\psi & sin\phi sin\theta cos\psi -cos\phi sin\psi \; & cos\phi sin\theta cos\psi +sin\phi sin\psi \\ cos\theta sin\psi & sin\phi sin\theta sin\psi +cos\phi cos\psi \; & cos\phi sin\theta sin\psi -sin\phi cos\psi \\ sin\theta & -sin\phi cos\theta & -cos\phi cos\theta \end{array}\right],$$

$$Q_{2}=\left[\begin{array}{c} -g\sin\theta +\frac{\rho V_{*}^{2}}{2m}S\left[C_{X}\left(\alpha \right)+C_{X_{q}}\left(\alpha \right)\frac{cq}{2V_{*}}+C_{X_{\delta_{e}}}\left(\alpha \right)\right]+\frac{\rho S_{p}C_{p}}{2m}\left[{\left(k_{M}\delta_{t}\right)}^{2}-V_{*}^{2}\right]\\ g\sin\phi \cos\theta +\frac{\rho V_{*}^{2}}{2m}S\left[C_{Y_{0}}+C_{Y_{\beta }}+C_{Y_{p}}\frac{bp}{2V_{*}}+C_{Y_{r}}\frac{br}{2V_{*}}+C_{Y_{\delta_{a}}}\delta_{a}\right]\\ g\cos\phi \cos\theta +\frac{\rho V_{*}^{2}}{2m}S\left[C_{Z}\left(\alpha \right)+\right.\left.C_{Z_{q}}\left(\alpha \right)+C_{Y_{p}}\frac{cq}{2V_{*}}+C_{Z_{\delta_{e}}}\left(\alpha \right)\delta_{e}\right] \end{array}\right],$$

$$Q_{3}=\left[\begin{array}{ccc} 1 & \sin\phi \tan\theta & \cos\phi \tan\theta \\ 0 & \cos\phi & -\sin\phi \\ 0 & \frac{\sin\phi }{\cos\theta } & \frac{\cos\phi }{\cos\theta } \end{array}\right],$$

$$Q_{4}=\left[\begin{array}{c} \frac{I_{xz}\left(I_{x}-I_{y}+I_{z}\right)}{I_{x}I_{z}-I_{xz}^{2}}pq-\frac{I_{z}\left(I_{z}-I_{y}\right)+I_{xz}^{2}}{I_{x}I_{z}-I_{xz}^{2}}qr\\ \frac{I_{z}-I_{x}}{I_{y}}pr-I_{x}I_{y}pr-\frac{I_{xz}}{I_{y}}\left(p^{2}-r^{2}\right)\\ \frac{\left(I_{x}-I_{y}\right)I_{x}+I_{xz}^{2}}{I_{x}I_{z}-I_{xz}^{2}}pq-\frac{I_{xz}\left(I_{x}-I_{y}+I_{z}\right)}{I_{x}I_{z}-I_{xz}^{2}}qr \end{array}\right],$$

$$\begin{array}{c} Q_{5}=\left[\begin{array}{c} \frac{\rho V_{a}^{2}bS}{2}C_{l_{0}}+C_{l_{\beta }}\beta +\left[C_{l_{p}}\frac{b}{2V_{*}}p+C_{l_{r}}\frac{b}{2V_{*}}r+C_{l_{\delta_{a}}}\delta_{a}\right]+\ldots \\ \frac{\rho V_{a}^{2}Sc}{2}[C_{m_{0}}+C_{m_{\alpha }}\alpha +\ldots \\ \frac{\rho V_{*}^{2}bS}{2}\left[C_{l_{0}}+C_{l_{\beta }}\beta +C_{l_{p}}\frac{b}{2V_{*}}p+C_{l_{r}}\frac{b}{2V_{*}}r+C_{l_{\delta_{a}}}\delta_{a}\right]+\ldots \end{array}\right.\\ \left.\begin{array}{c} \ldots +\frac{\rho V_{a}^{2}bS}{2}\left[C_{n_{0}}C_{n_{\beta }}\beta +C_{n_{p}}\frac{b}{2V_{*}}p+C_{n_{r}}\frac{b}{2V_{*}}r+C_{n_{\delta_{a}}}\delta_{a}\right]\\ \ldots +C_{m_{q}}\frac{c}{2V_{*}}q+C_{m_{\delta_{e}}}\delta_{e}]\\ \ldots +\frac{\rho V_{*}^{2}bS}{2}\left[C_{n_{0}}+\right.\left.C_{n_{\beta }}\beta +C_{n_{p}}\frac{b}{2V_{*}}p+C_{n_{r}}\frac{b}{2V_{*}}r+C_{n_{\delta_{a}}}\delta_{a}\right] \end{array}\right], \end{array}$$

**Figure 2:**
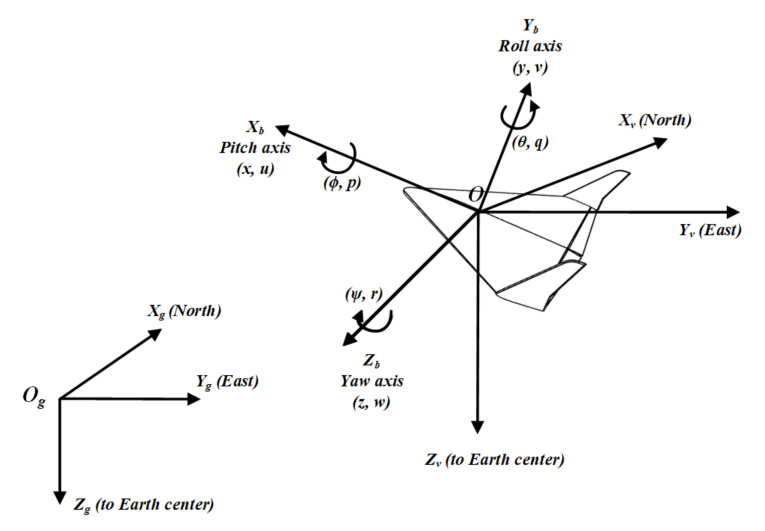
Definitions of coordinate frames.

$C_{X_{\alpha }}=-C_{D_{\alpha }}\cos\alpha +C_{L_{\alpha }}\sin\alpha$,

$C_{X_{q}}=-C_{D_{q}}\cos\alpha +C_{L_{q}}\sin\alpha$,

$C_{X_{\delta_{e}}}=-C_{D_{\delta_{e}}}\cos\alpha +C_{L_{\delta_{e}}}\sin\alpha$,

$C_{Z_{\alpha }}=-C_{D_{\alpha }}\sin\alpha -C_{L_{\alpha }}\cos\alpha$,

$C_{Z_{q}}=-C_{D_{q}}\sin\alpha -C_{L_{q}}\cos\alpha$,

$C_{Z_{\delta_{e}}}=-C_{D_{\delta_{e}}}\sin\alpha -C_{L_{\delta_{e}}}\cos\alpha$,

$C_{X_{\alpha }},C_{X_{q}},C_{X_{\delta_{e}}},C_{Z_{\alpha }},C_{Z_{q}},C_{Z_{\delta_{e}}}$—nonlinear functions of angle of attack;

*x*—inertial North UAV position in the **O**${}_{g}$**X**${}_{g}$**Y**${}_{g}$**Z**${}_{g}$ (Figure 2);

*y*—inertial East UAV position in the **O**${}_{g}$**X**${}_{g}$**Y**${}_{g}$**Z**${}_{g}$ (Figure 2);

*z*—inertial Down position in ***O${}_{g}$X${}_{g}$Y${}_{g}$Z${}_{g}$*** (Figure 2);

*ϕ, θ, ψ*—roll angle, pitch angle, yaw angle respectively;

*u*, *v*, *w*—body frame velocities measured in ***OX${}_{b}$Y${}_{b}$Z${}_{b}$*** (Figure 2);

*p*, *q*, *r*—angular rates measured in ***OX${}_{b}$Y${}_{b}$Z${}_{b}$*** (roll rate, pitch rate, yaw rate respectively);

***OX${}_{v}$Y${}_{v}$Z${}_{v}$***—MAVvehicle frame with origin in the MAV center of mass, axes are aligned with ***O${}_{g}$X${}_{g}$Y${}_{g}$Z${}_{g}$*** axis;

C${}_{*}$—aerodynamic coefficients (values in a Table 2 and Table 3);

δ${}_{a}$, δ${}_{e}$, δ${}_{t}$—aileron, elevator, throttle commands;

V${}_{*}$—airspeed;

β—sideslip angle.

### 2.3. MAV Simulation System

**Figure 3:**
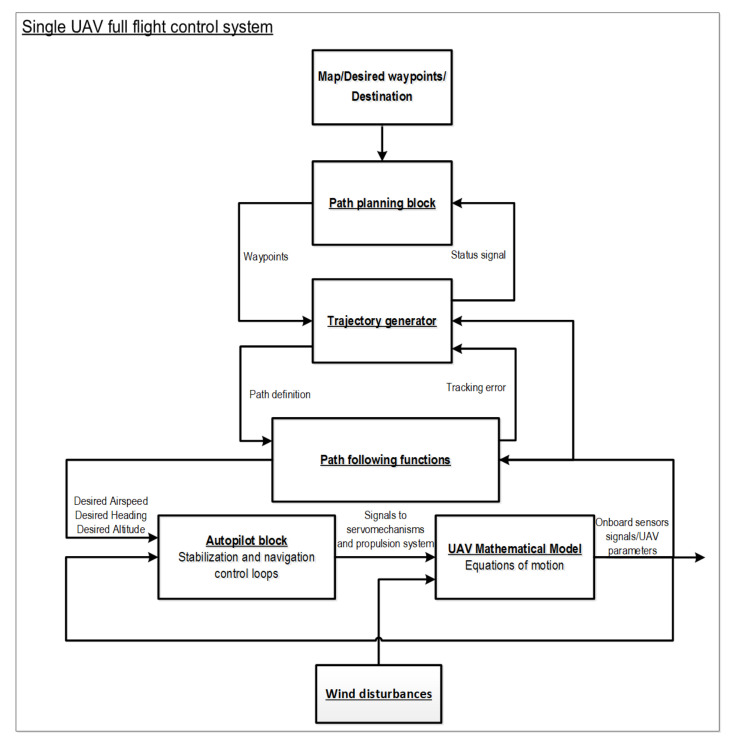
Single UAV control system architecture.

**Figure 4:**
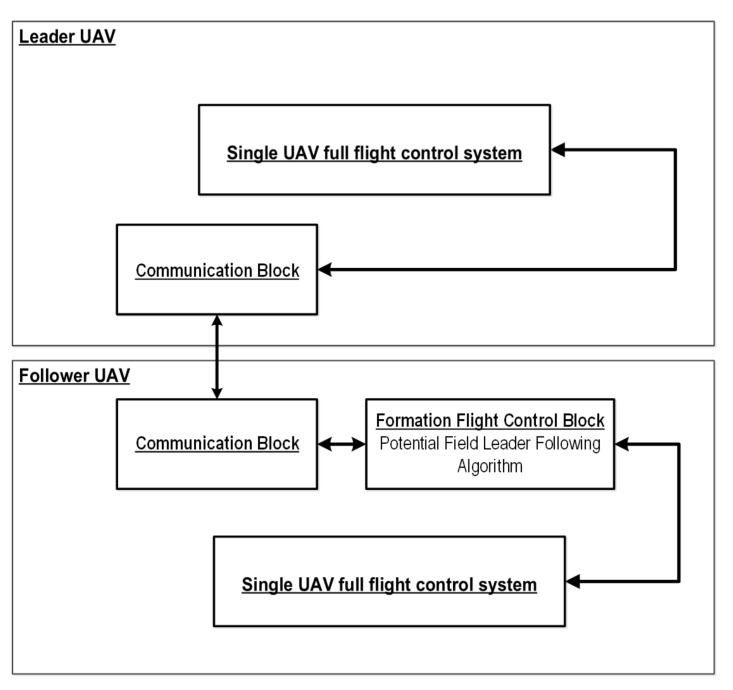
Simulation system architecture.

The 12 nonlinear, coupled, first-order, ordinary differential Equations (2)–(5) represent the mathematical model of the MAV. Based on these equations, a simulation model was constructed in Matlab/Simulink software using specialized toolboxes like Control System [70], Flight Dynamics and Control [71], and Aerospace [72]. Presented Equations (2)–(5) create the MAV’s kinematics and dynamics block (Figure 3). Additionally, an autopilot model whose primary goal is to control the MAV’s inertial position and attitude was also developed. Apart from stabilization and navigation control loops, the developed simulation model of the Bullit 60 includes formation flight control loops, path following and path planning functions as well as a trajectory generator block (Figure 3). Path-following functions generate commands to low-level control loops (stabilization and navigation control loops). The path planning block produces a sequence of straight-line paths that maneuver the MAV. The block labeled as the trajectory generator is responsible for switching between orbit and straight-line path following to maneuver the MAV along defined waypoints on the terrain map. The prepared simulation model provides a full flight MAV control system during various phases of flight and emulates commercial autopilots like MicroPilot [73] or Kestrel [74]. Simulation systems utilized during the MAV formation flight control tests were composed of two separate MAV blocks with autopilots (a full flight control system like the one presented in Figure 3): communication block which is responsible for data exchange between UAVs and a formation flight control block implementing a potential field leader following algorithm. The simulation model also takes into account positioning errors and noise in this process (not only the size of the GPS error but also the dynamic characteristics of the error. The GPS error model (both altitude and x-y plane) includes a slowly varying, zero-mean bias (4.7 m for x and y direction, 9.2 m for altitude) and a random noise component (0.4 m for x and y direction, 0.7 m for altitude). The model of the transient behavior of the error was approximated using the Gauss–Markov process based on [75] with a standard deviation of 0.21 m in x and y directions and of 0.4 m in altitude. Moreover, the simulation model also took into account atmospheric disturbances. The total wind vector was represented as a constant vector (steady ambient wind) and stochastic process (wind gusts and atmospheric disturbances). A suitable approximation of the non-steady gusts was given by Dryden transfer functions with turbulence intensities along vehicle frames x, y, z on level 2.12 m, 2.12 m and 1.4 m respectively for each direction [75]. A schematic of the entire system used within the study has been presented in Figure 4.

## 3. Virtual Electric Dipole Field for Autonomous Formation Flight

### 3.1. Formation Flight Geometry

**Figure 5:**
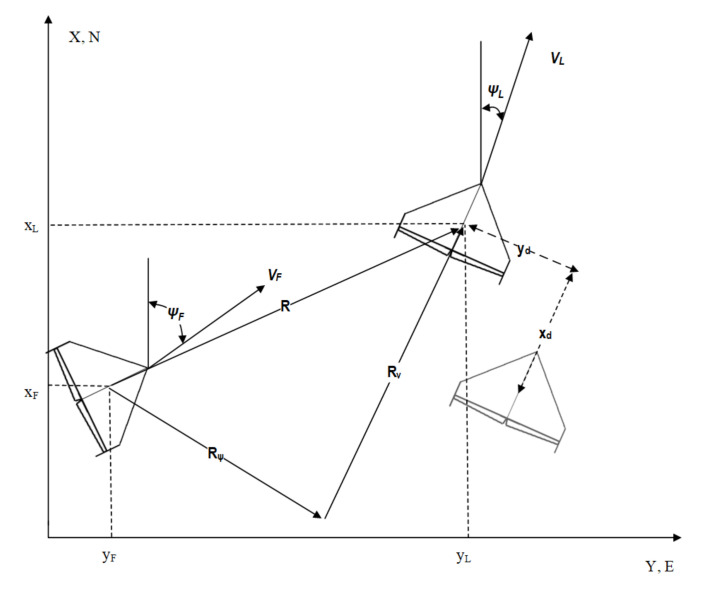
Formation flight geometry.

The formation flight control system is based on the leader–follower control strategy. The cosidered example concerns the case of two UAVs—one leader and one follower. This is due to the fact that future in-flight tests of the proposed formation flight algorithm will be carried out with a single leader–follower scheme on account of consideration for available hardware resources (autopilots, airframes, base stations, etc.). A simple formation geometry of two UAVs is presented in Figure 5. Leader and follower positions can be denoted with the following formulas:

(6) $$\begin{array}{c} L_{pos}=\left[\begin{array}{c} x_{L}\\ y_{L}\\ z_{L} \end{array}\right]\\ F_{pos}=\left[\begin{array}{c} x_{F}\\ y_{F}\\ z_{F} \end{array}\right] \end{array}.$$

The length of **R** (defined on the scheme in Figure 5) is defined as:

(7) $$∥R∥=\sqrt{∥R_{\psi }∥+∥R_{v}∥}.$$

The Follower UAV’s commanded trajectory can be defined as:

(8) $$T_{F}^{c}=\left[\begin{array}{c} V_{F}^{c}\\ \psi_{F}^{c}\\ A_{F}^{c} \end{array}\right],$$

where $V_{F}^{c}$, $\psi_{F}^{c}$, $A_{F}^{c}$ - is the follower’s desired airspeed, heading and altitude respectively.

### 3.2. Virtual Electric Dipole Field in Leader-Follower Control Structure

**Figure 6:**
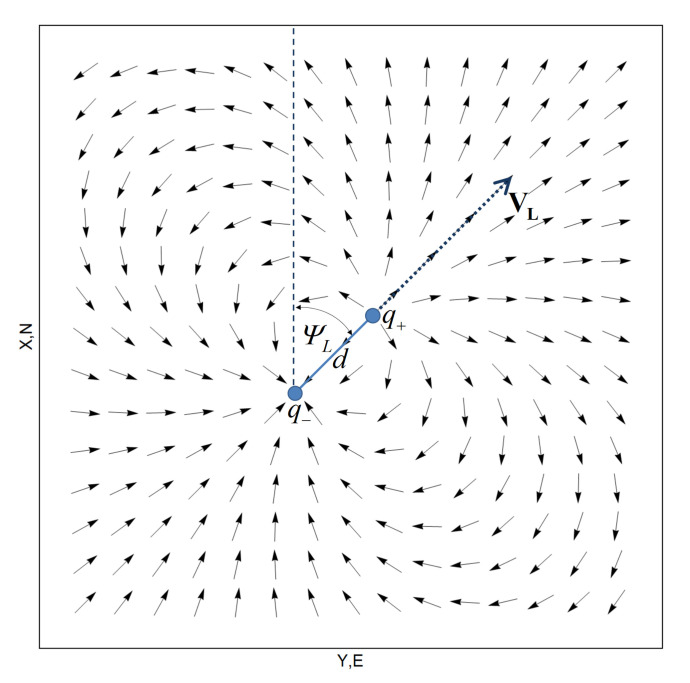
Electric dipole field.

The aim of the leader-follower formation control is to keep the follower in the desired position relative to the leader’s position. Additionally, when the follower reaches the desired position, its heading should be parallel to the leader’s heading. Therefore, the aim of formation flight control is to determine the heading and the velocity of the follower based on the leader’s position and velocity. For the purposes of this paper, the potential field method, utilized first by Khatib [76] to help mobile robots to avoid obstacles, was used to determine the follower’s heading angle. In later studies, the potential field method was used to plan the robot’s paths, see e.g., [77,78,79,80]. The main idea of the potential field method is to find a scalar field whose gradient will produce the desired vector field in space. This virtual vector field is then used in the control process. The vector field used to control the follower’s heading must have the following properties: lines of this field must meet at one point and reach this point in a manner that allows them to parallel the leader’s velocity. The electric dipole whose dipole moment vector forms angle $\psi_{L}$ (leader heading) with the x-axis and is, therefore, parallel to the longitudinal leader’s velocity **V**${}_{L}$, is a good candidate for the generation of such a field. The electric vector field generated by the electric dipole is shown in Figure 6.

The dipole electric field has the following features: the direction of the electric field is tangent to the field line at any point in space, the field lines begin on positive charge *q${}_{+}$* and terminate on negative charge *q${}_{-}$*, the field lines can never cross. When the direction of such an electric vector field defines the desired follower’s heading, the path of the follower’s UAV coincides with the field lines (the direction of the follower’s velocity is determined by its heading, and by definition, the velocity is always tangent to the path). It can therefore be said that the position of charge *q${}_{-}$* is an attractor for the follower, which ensures the stability of the system control. As can be seen in Figure 6, from almost any point within the space, the electric field lines reach point *q${}_{-\;}$* circling the dipole. There is an exception, however, the part of space between dipole charges. In the case when the point *q${}_{-}$* is the desired follower’s position and the direction of the electric vector field defines the desired follower’s heading, the follower reaches point *q${}_{-}$* circling it. This behavior is consistent with intuition: if the electric dipole is placed in the leader position and *q${}_{-}$* is the desired follower’s position, the follower should just go around the leader and position behind him.

**Figure 7:**
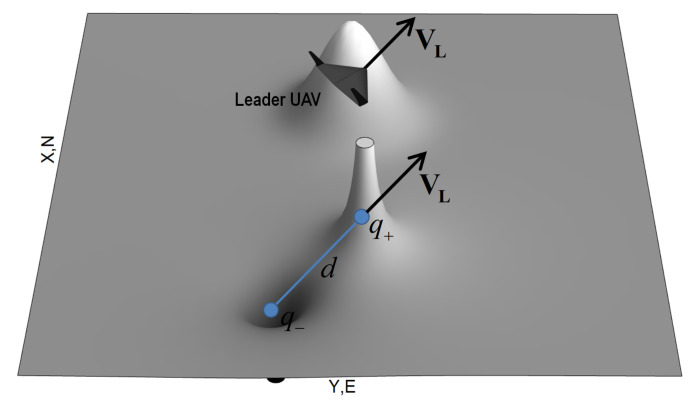
Lider-dipole potential field.

The follower’s desired position relative to the leader’s position can be arbitrary (the follower on the leader’s right side, for example) and the virtual electric dipole position can also be arbitrary. The potential field of an exemplary leader-dipole configuration is shown in Figure 7.

Figure 7 also presents an additional potential field in the leader’s position. This extra field creates a collision-preventing area protecting the leader from colliding with the follower.

### 3.3. Potential Field Generation and Desired Heading Definition in Formation Flight

Let the leader’s position in the global frame in the plane YX be described by vector:

(9) $$R_{L}=\left(y_{L},x_{L}\right),$$

and the desired follower‘s position relative to the leader in the global frame by vector:

(10) $$R_{d}=\left(x_{d}\sin\left(\psi_{L}\right)+y_{d}\cos\left(\psi_{L}\right),x_{d}\cos\left(\psi_{L}\right)-y_{d}\sin\left(\psi_{L}\right)\right),$$

where $\left(y_{d},x_{d}\right)$—follower’s desired position relative to the leader in the leader’s body frame (please see Figure 5).

The follower’s desired position in the global frame is described by vector:

(11) $$R_{dF}=R_{L}+R_{d}.$$

The electric field of the dipole has two singular points—these are points where electrical charges are placed. These are critical because within their proximity the vector field can rapidly change direction by as much as 180 degrees, changing the desired heading just as rapidly. The follower’s desired position must not be placed at these critical points. The position of the negative charge of the electric dipole in the global frame is therefore described by the vector:

(12) $$R_{-}=R_{dF}+a\left(\sin\left(\psi_{L}\right),\cos\left(\psi_{L}\right)\right).$$

**Figure 8:**
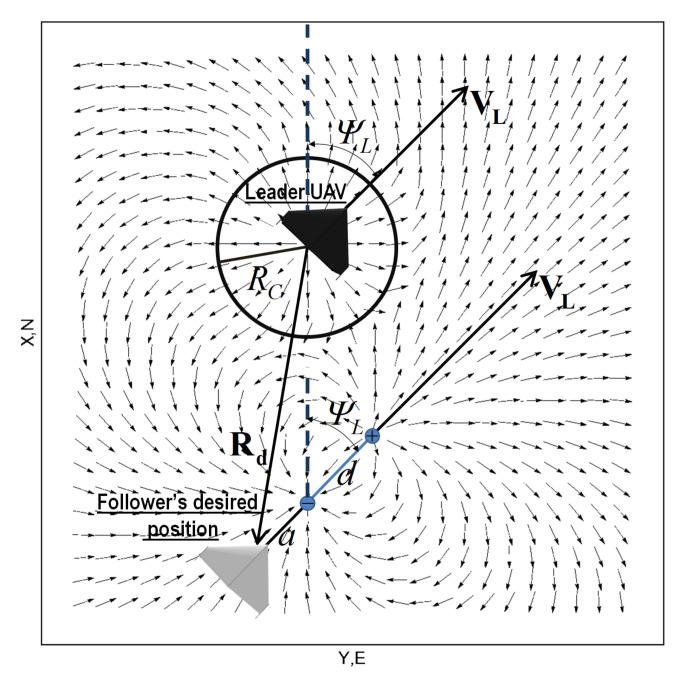
Exemplary vector field setting out the follower’s heading.

As can be seen in Equation (12), the position of the dipole’s negative charge does not correspond to the follower’s desired position (Equation (11)). Rather, the dipole’s negative charge position is shifted in the direction of the leader’s $\psi_{L}$ heading by a value of *a* (see Figure 8). Position of the positive charge of the electric dipole in the global frame is described by a vector:

(13) $$R_{+}=R_{dF}+\left(d+a\right)\left(\sin\left(\psi_{L}\right),\cos\left(\psi_{L}\right)\right).$$

Based on the positions of the virtual electric charges (Equations (12) and (13)), it becomes possible to determine the potential field. A potential field generated by a positive charge at $\left(y,x\right)$ position is presented below:

(14) $$V_{+}=\frac{q_{c}}{∥R_{R+}∥},$$

where: $R_{R+}=\left(y,x\right)-R_{+}$, $q_{c}$—is the electric charge value.

A negative charge generates the following potential field:

(15) $$V_{-}=\frac{-q_{c}}{∥R_{R-}∥},$$

where $R_{R-}=\left(y,x\right)-R_{-}$.

A potential field protecting the leader against a collision with the follower is expressed as:

(16) $$V_{C}=q_{c}\;exp^{\frac{-R_{RC}{}^{2}}{C_{RC}R_{C}{}^{2}}}$$

where $R_{RC}=\left(y,x\right)-R_{L}$, $R_{C}$ is the collision’s radius, $C_{RC}$ is the collision’s coefficient.

When $R_{RC}>R_{C}$, this protection field should disappear. It can be assumed that this happens in situations where $V_{C}$ is equal to 0.01 of its maximum value (equal to $q_{c}$). Under this assumption the collision’s coefficient $C_{RC}$ is equal to 0.217.

According to Equations (16) and (17) the total potential field at point $\left(y,x\right)$ takes the form of:

(17) $$V_{T}=V_{+}+V_{-}+V_{C}$$

Taking into account Equations (9)–(16), Equation (17) can be written as:

(18) $$\begin{array}{c} V_{T}=-\frac{q_{c}}{\sqrt{{\left(a\cos\left(\psi_{L}\right)+x_{d}\cos\left(\psi_{L}\right)-y_{d}\sin\left(\psi_{L}\right)+x_{L}-x\right)}^{2}+{\left(a\sin\left(\psi_{L}\right)+x_{d}\sin\left(\psi_{L}\right)+y_{d}\cos\left(\psi_{L}\right)+y_{L}-y\right)}^{2}}}+\\ \frac{q_{c}}{\sqrt{{\left(\left(a+d\right)\cos\left(\psi_{L}\right)+x_{d}\cos\left(\psi_{L}\right)-y_{d}\sin\left(\psi_{L}\right)+x_{L}-x\right)}^{2}+{\left(\left(a+d\right)\sin\left(\psi_{L}\right)+x_{d}\sin\left(\psi_{L}\right)+y_{d}\cos\left(\psi_{L}\right)+y_{L}-y\right)}^{2}}}+\\ q_{c}\;exp^{-\frac{{\left(x-x_{L}\right)}^{2}+{\left(y-y_{L}\right)}^{2}}{R_{C}^{2}C_{RC}}} \end{array}$$

The next step is to determine the vector field based on the scalar potential field (18). The vector field for controlling follower’s heading is determined using the following formula:

(19) $$E_{T}=\left(E_{y},E_{x}\right)=-grad\;V_{T}$$

Taking into account Equations (18) and (19), components of the vector field take the form of:

(20) $$\begin{array}{c} E_{y}=\frac{q_{c}\left(a\sin\left(\psi_{L}\right)+x_{d}\sin\left(\psi_{L}\right)+y_{d}\cos\left(\psi_{L}\right)+y_{L}-y\right)}{{\left({\left(a\cos\left(\psi_{L}\right)+x_{d}\cos\left(\psi_{L}\right)-y_{d}\sin\left(\psi_{L}\right)+x_{L}-x\right)}^{2}+{\left(a\sin\left(\psi_{L}\right)+x_{d}\sin\left(\psi_{L}\right)+y_{d}\cos\left(\psi_{L}\right)+y_{L}-y\right)}^{2}\right)}^{3/2}}-\\ \frac{q_{c}\left(\left(a+d\right)\sin\left(\psi_{L}\right)+x_{d}\sin\left(\psi_{L}\right)+y_{d}\cos\left(\psi_{L}\right)+y_{L}-y\right)}{{\left({\left(\left(a+d\right)\cos\left(\psi_{L}\right)+x_{d}\cos\left(\psi_{L}\right)-y_{d}\sin\left(\psi_{L}\right)+x_{L}-x\right)}^{2}+{\left(\left(a+d\right)\sin\left(\psi_{L}\right)+x_{d}\sin\left(\psi_{L}\right)+y_{d}\cos\left(\psi_{L}\right)+y_{L}-y\right)}^{2}\right)}^{3/2}}+\\ \frac{2q_{c}\left(y-y_{L}\right)e^{-\frac{{\left(x-x_{L}\right)}^{2}+{\left(y-y_{L}\right)}^{2}}{R_{C}^{2}C_{RC}}}}{R_{C}^{2}C_{RC}}\\ E_{x}=\frac{q_{c}\left(a\cos\left(\psi_{L}\right)+x_{d}\cos\left(\psi_{L}\right)-y_{d}\sin\left(\psi_{L}\right)+x_{L}-x\right)}{{\left({\left(a\cos\left(\psi_{L}\right)+x_{d}\cos\left(\psi_{L}\right)-y_{d}\sin\left(\psi_{L}\right)+x_{L}-x\right)}^{2}+{\left(a\sin\left(\psi_{L}\right)+x_{d}\sin\left(\psi_{L}\right)+y_{d}\cos\left(\psi_{L}\right)+y_{L}-y\right)}^{2}\right)}^{3/2}}-\\ \frac{q_{c}\left(\left(a+d\right)\cos\left(\psi_{L}\right)+x_{d}\cos\left(\psi_{L}\right)-y_{d}\sin\left(\psi_{L}\right)+x_{L}-x\right)}{{\left({\left(\left(a+d\right)\cos\left(\psi_{L}\right)+x_{d}\cos\left(\psi_{L}\right)-y_{d}\sin\left(\psi_{L}\right)+x_{L}-x\right)}^{2}+{\left(\left(a+d\right)\sin\left(\psi_{L}\right)+x_{d}\sin\left(\psi_{L}\right)+y_{d}\cos\left(\psi_{L}\right)+y_{L}-y\right)}^{2}\right)}^{3/2}}+\\ \frac{2q_{c}\left(x-x_{L}\right)e^{-\frac{{\left(x-x_{L}\right)}^{2}+{\left(y-y_{L}\right)}^{2}}{R_{C}^{2}C_{RC}}}}{R_{C}^{2}C_{RC}} \end{array}$$

The vector field $E_{T}$ has been used to control the follower’s heading. Finally, the angle between vector $E_{T}$ and the positive X,N-axis in the follower’s position define the follower’s desired heading:

(21) $$\psi_{F}^{c}=∠\left(X,N,\;\;\;E_{T}\right).$$

An exemplary vector field setting out the follower’s heading is shown in Figure 8. In conclusion, it should be emphasized that the proposed vector field has one big advantage: a lack of local minima and only one global minimum in the position of the negative charge (minus infinity). The absence of additional minima makes control processes using such potential fields reliable because such control systems have only one attractor.

### 3.4. Velocity Control in Formation Flight

Realization of effective formation flights also requires precise control over the follower UAV’s velocity. In the approach presented within this work, velocity control is based on position errors between the leader and the follower. These position errors can be defined as follows:

(22) $$e_{xyz}=L_{pos}-F_{pos}=\left[\begin{array}{c} x_{L}\\ y_{L}\\ z_{L} \end{array}\right]-\left[\begin{array}{c} x_{F}\\ y_{F}\\ z_{F} \end{array}\right]=\left[\begin{array}{c} e_{x}\\ e_{y}\\ e_{z} \end{array}\right].$$

Errors defined in Equation (22) need to be expressed in the formation frame. In the formation frame they can be written as follows:

(23) $$∥R_{v}∥=\sin\psi_{L}e_{x}+\cos\psi_{L}e_{y},$$

(24) $$∥R_{\psi }∥=\cos\psi_{L}e_{x}-\sin\psi_{L}e_{y}.$$

Position errors expressed in the formation frame in x and y directions are defined as $∥R_{v}∥$ (23) and $∥R_{\psi }∥$ (24). To control the follower’s velocity during formation flight only the $R_{v}$ component of the vector *R* is used. The position error $∥R_{v}∥$ expressed in the formation frame defined with Equation (23) should contain the desired distance in direction x designated as $x_{d}$. This can be expressed in the following manner:

(25) $$e_{v}=∥R_{v}∥-x_{d}=\left[\sin\psi_{L}e_{x}+\cos\psi_{L}e_{y}\right]-x_{d}.$$

**Figure 9:**
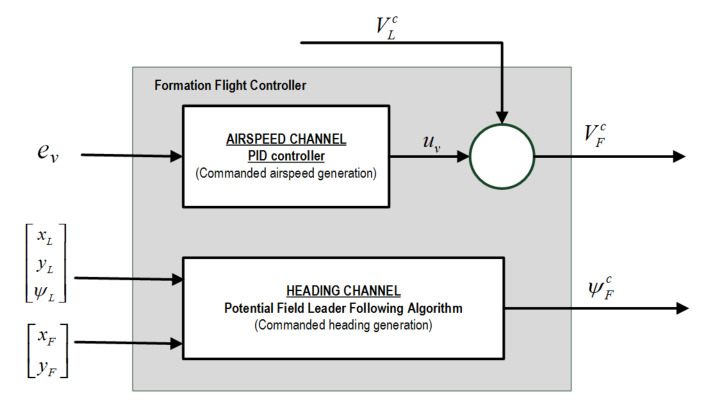
Formation flight controller scheme.

Parameter $e_{v}$ is the input signal to the velocity controller (please refer to scheme in Figure 9). Discrete time forms of the controller for producing airspeed corrections are presented below:

(26) $$u_{v}=k_{p}+k_{d}\frac{N}{1+N\frac{T_{s}}{2}\frac{Z+1}{Z-1}},$$

where *k${}_{p}$*, *k${}_{d}$* are controller gains, *T${}_{s}$* is sampling time, *N* is the filter coefficient, Z is a discrete time operator from Z transform.

During formation flight control the desired altitude signal for the follower UAV is produced in accordance with the following equation:

(27) $$A_{F}^{c}=A_{L}^{c}+z_{d},$$

where $z_{d}$ is the desired separation distance in direction *z*.

## 4. Results

Simulation tests of the proposed formation flight control algorithm were performed with one leader and one follower. The leader had information about the desired trajectory. The follower’s task was to follow the leader using the potential field algorithm. Performed tests were done using straight-line path following as well as the loiter function (following a circular path with a specified radius). Initial parameters (positions, headings) of the leader and the follower were different and were changed during the tests (please refer to Table 4 and Table 5). Other parameters utilized in the Equation (20) were: *R${}_{C}$* = 20 m, *C${}_{RC}$* = 0.217, *a* = 20 m, *d* = 20 m, $q_{c}$ = 1. During simulation tests MAVs dynamics were influenced by atmospheric disturbances **$V_{{}_{wind}}=V_{w_{{}_{const}}}+V_{w_{dyn}}$** modeled as constant wind field **$V_{w_{{}_{const}}}={\left[\begin{array}{ccc} w_{x} & w_{y} & w_{z} \end{array}\right]}^{T}$**(where **$w_{x}$ = **1 m/s, **$w_{y}$ = **3 m/s, **$w_{z}$ = **0 m/s ) expressed in the inertial frame with added turbulences **$V_{w_{dyn}}$** (non-steady gusts). The turbulences **$V_{w_{dyn}}$** were generated by passing white noise signal through Dryden transfer functions. Dryden gust model parameters were taken for low altitudes and moderate turbulences in accordance to [64]. Simulation studies include seven test flights (four tests for straight-line and three for circle path).

### 4.1. Straight Line Path Following Tests

**Figure 10:**
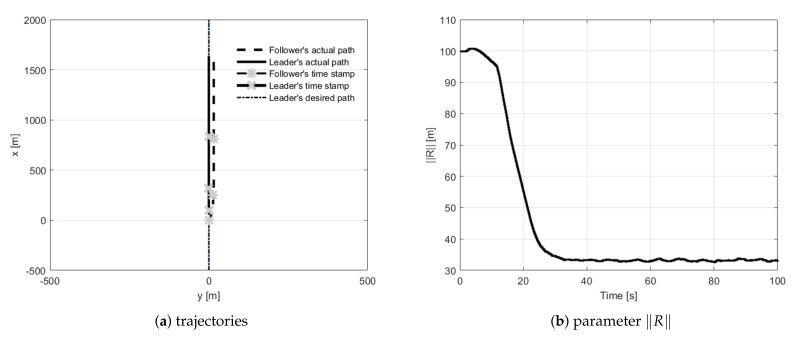
UAVs positions during formation flight for Test 1: (**a**) trajectories, (**b**) parameter $∥R∥$.

**Figure 11:**
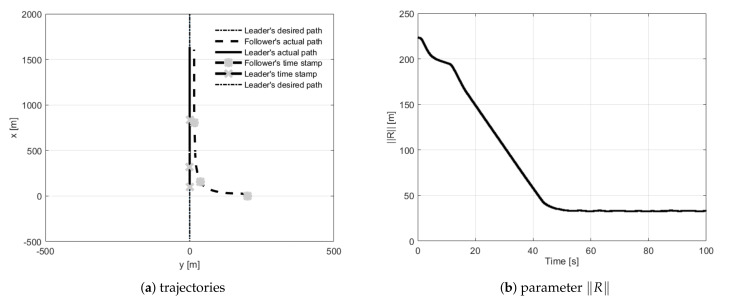
UAVs positions during formation flight for Test 2: (**a**) trajectories, (**b**) parameter $∥R∥$.

**Figure 12:**
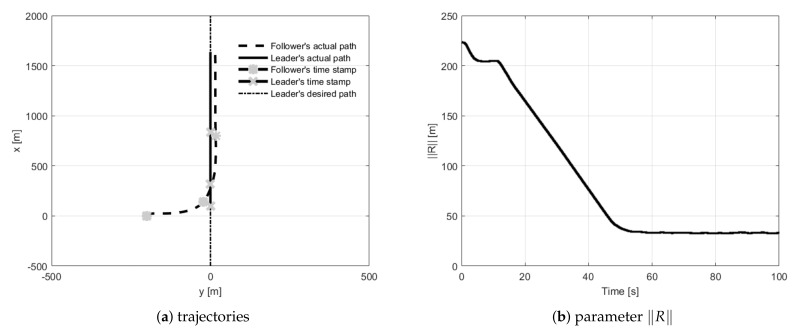
UAVs positions during formation flight for Test 3: (**a**) trajectories, (**b**) parameter $∥R∥$.

**Figure 13:**
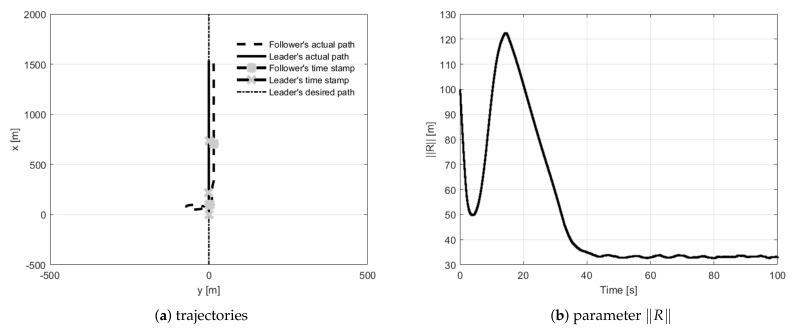
UAVs positions during formation flight for Test 4: (**a**) trajectories, (**b**) parameter $∥R∥$.

**Figure 14:**
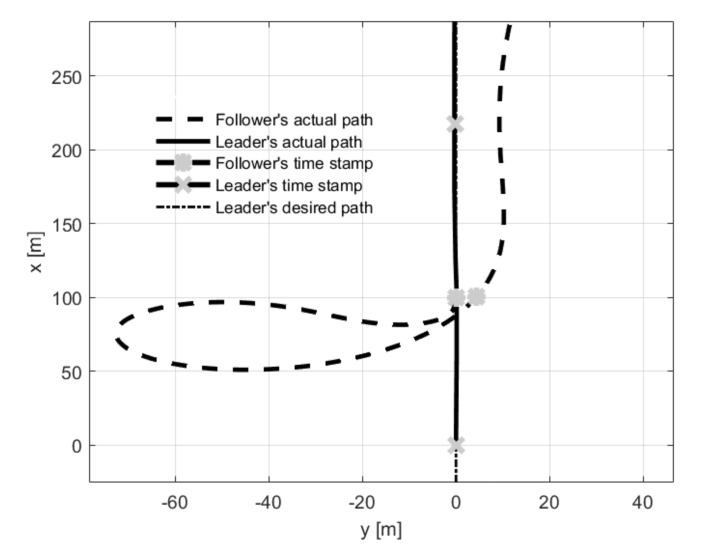
Test 4: leader’s and follower’s trajectories during formation flight-zoom in.

During straight-line path following tests, the follower had to fly 30 m behind the leader and 15 m to its left side. The first trial concerned the situation when the follower’s initial position was placed 100 m behind the leader (Figure 10). During trials, two and three, the follower’s initial position was set 200 m to the left of and to the right of the leader (Figure 11 and Figure 12). The fourth test concerned the situation when the follower’s initial position was in front of the leader with the two given opposite headings to provoke a potential collision (Figure 13 and Figure 14). During all tests vector **R** was measured and presented to confirm the quality of the formation flight control system.

### 4.2. Circle Path Following Tests

**Figure 15:**
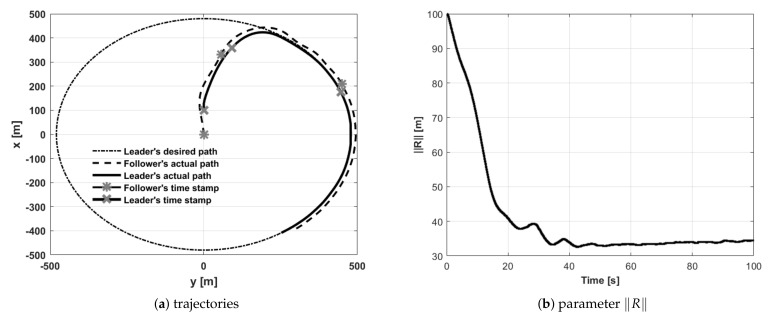
UAVs positions during formation flight for Test 5: (**a**) trajectories, (**b**) parameter $∥R∥$.

The second portion of simulation tests was associated with following a circular path. The first loitering trial concerned a situation where the follower had to fly behind the leader at a distance of 30 m as well as 15 m to its left. Initial headings of the leader and the follower were the same and were equal to 0 rad. The leader’s initial position was in front of the follower. Figure 15 shows the paths of the leader and the follower during trial 5. To show the quality of formation flight control, the length of vector **R** has also been presented. Test 5 concerned a case when the follower’s initial position was behind the leader (with both having the same initial headings). The follower’s behavior could easily be predicted—the follower caught up with the leader and then fell into the desired position.

**Figure 16:**
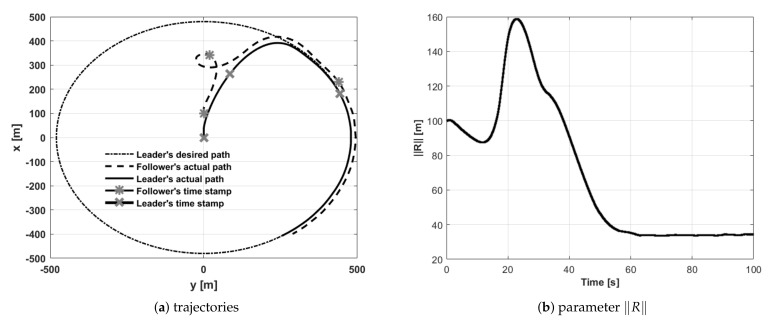
UAVs positions during formation flight for Test 6: (**a**) trajectories, (**b**) parameter $∥R∥$.

**Figure 17:**
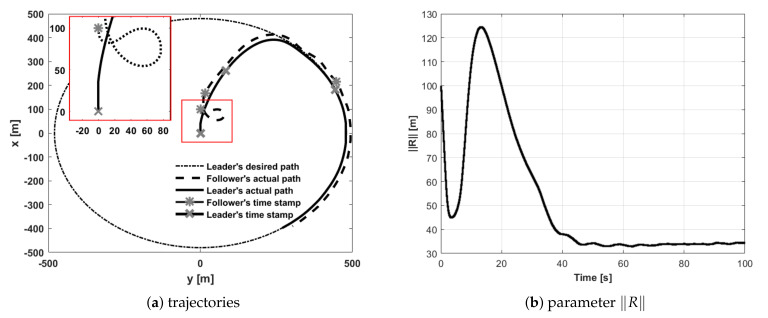
UAVs positions during formation flight for Test 7: (**a**) trajectories, (**b**) parameter $∥R∥$.

**Figure 18:**
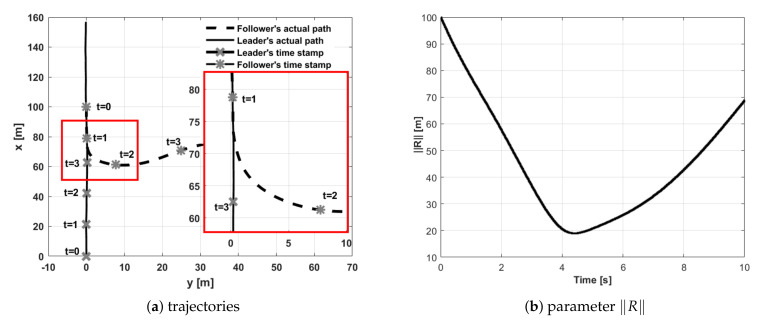
UAVs positions during test for Test 7—collision avoidance: (**a**) trajectories, (**b**) parameter $∥R∥$.

**Figure 19:**
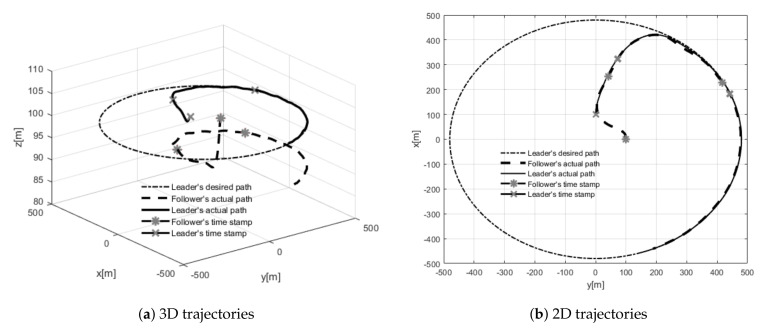
UAVs positions during test for Test 8: (**a**) 3D trajectories, (**b**) 2D trajectories.

**Figure 20:**
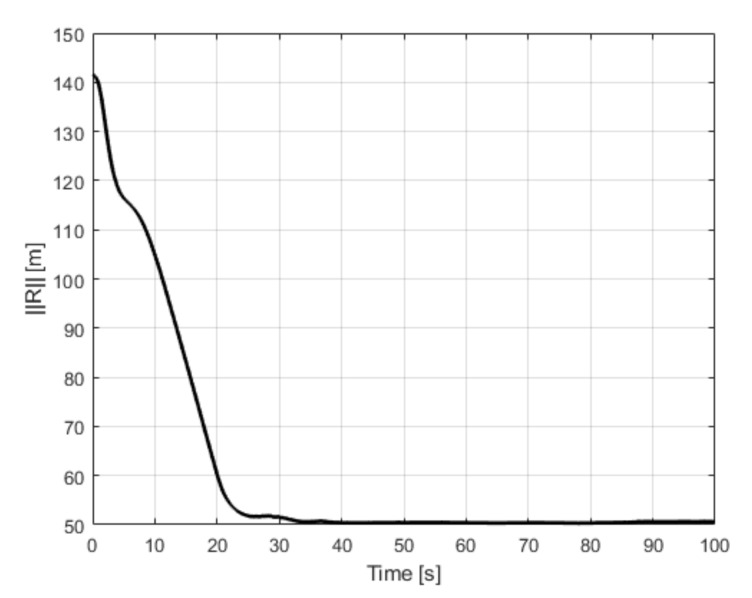
UAVs positions during test for Test 8: parameter $∥R∥$.

Test number 6 concerned a situation when the leader’s initial position was behind the follower (with both having the same desired separation distance). Results of this attempt are shown in Figure 16. The follower’s heading is determined by an electric vector field generated by a virtual electric dipole (see Figure 8), so at the beginning, the follower turned left, next it made a circle and settled behind the leader in the desired position. This behavior is consistent with one’s intuition. Test number 7 presented a case where the leader’s and follower’s initial headings were opposite. Figure 17 and Figure 18 show the results of this test. In this case, the follower’s behavior is similar to test 6—the follower first turned left and next made a circle. For the circle trajectory following, an additional test (number 8) was performed in which the desired altitude of the leader and follower were different, while the follower follows the leader without position shifting respect the leader on the x–y plane. The results are presented in the form of a 3D trajectory and its projection on the x–y plane (Figure 19 as well as a time course of the $∥R∥$ parameter (Figure 20).

Simulation tests also verified the collision avoidance efficiency of the proposed algorithm. In Equation (17) the part related to the formation flight was turned off ($V_{+}=V_{-}=0$). Only the component responsible for collision avoidance was left active. The leader and the follower were installed 100 m apart and given opposite headings (0 rad and π rad). Both UAVs had to follow the same straight line to create a likelihood of a collision situation. The results of this test have been presented in the Figure 13, Figure 17 and Figure 18. An analysis of the follower’s trajectory shows no collision occurred. The follower deviated from its established path and avoided a collision with the second UAV. During the test, the $∥R∥$ parameter value decreased to about 20 m indicating that distance as the closest proximity of the UAVs. Furthermore, this distance can be modified through parameter *R${}_{C}$.* The most convenient way to check the quality of the proposed algorithm is to determine some statistical parameters. For flight tests 1– 7, the root mean square error (**RMSE**) and relative root mean square error (**RRMSE**) between the desired and the real follower’s position were determined. These indicators were calculated for the $∥R∥$ parameter logged during 100 sec long simulations. For RMSE and RRMSE calculations, only the last 30sec of data were used to ensure that the transient process expired and both UAVs positions were set properly. Established indicators have been presented in Table 6.

Concerning the existence of random wind, the accuracy of the MAV model, quality of the composed simulation system, and the leader’s nonlinear motion (circular path following) an error of approximately 30 cm can be considered to be a satisfactory result. The influence of random wind and inaccuracies of GNSS measurements can also be observed in figures labeled as “parameter $∥R∥$ during flight” where the parameter R performs small oscillations. Additionally, during the simulation tests, the impact of the communication delay between leader and follower was checked. During the straight-line path following the communication delay was changed from 0.1 s to 2.0 s and the RMSE and RRMSE were calculated from the last 30 s of 100 s long simulations (Table 7). It can be easily noticed that the RMSE and RRMSE parameters grow along with a rise in the time delay. In the case of the circle path following the communication delay over 2 s was a critical value and caused the follower not to reach its desired position behind the leader.

## 5. Conclusions

The paper presents a new method for controlling the formation flight of Unmanned Aerial Vehicles (UAVs). The control algorithm proposed within the work uses a virtual electric dipole field to determine a desired heading for the follower UAV. Furthermore, the manner of potential field generation provides protection from collisions during operations employing multiple UAVs. Simulation results presented in Chapter 4 are promising. A major advantage of using an electric field generated by a virtual electric dipole is its universality, reliability, and the fact that the follower’s heading is determined directly and not as corrections from the controller. This new method eliminates the need for a mixed control strategy since it works correctly within the entire phase space of the leader-follower system. One disadvantage of the presented method might be the computational complexity of the control algorithm. However, it should be noted that the computational resources of UAVs are constantly growing, and small, lightweight, high-performance on-module computers with low power consumption are available. Yet another disadvantage of the proposed system is that the follower’s velocity control strategy is different from its heading control. Achievement of a unified system for the control of the follower’s heading and velocity will be the subject of further research. The authors also plan to expand research into systems with more than one follower. Possibilities for changing formation flight hierarchy (where the leader becomes the follower and the follower becomes the leader) may also become the focus of future studies. It is worth mentioning that the described algorithm was successfully verified during in-flight tests, the results of which will be presented in the following article.

## Figures and Tables

**Table 1:** Bullit 60 moments and products of inertia.

Moment of Inertia	Value
*I${}_{x}$*	0.091108 [kgm${}^{2}$]
*I${}_{y}$*	0.076144 [kgm${}^{2}$]
*I${}_{z}$*	0.165955 [kgm${}^{2}$]
*I${}_{xz}$*	0.0011547 [kgm${}^{2}$]

**Table 2:** The Bullit logitudinal aerodynamic coefficients.

$C_{L_{0}}$	$C_{D_{0}}$	$C_{m_{0}}$	$C_{L_{\alpha }}$	$C_{D_{\alpha }}$	$C_{m_{\alpha }}$
0.14635	0.00293	−0.030586	2.8074	0.08759	−0.59632
$C_{L_{q}}$	$C_{D_{q}}$	$C_{m_{q}}$	$C_{L_{\delta_{e}}}$	$C_{D_{\delta_{e}}}$	$C_{m_{\delta_{e}}}$
4.0992	0.12967	−1.6137	1.05	0.0338	−0.61558

**Table 3:** The Bullit lateral aerodynamic coefficients.

$C_{Y_{0}}$	$C_{l_{0}}$	$C_{n_{0}}$	$C_{Y_{\beta }}$	$C_{l_{\beta }}$
−6.6478 × 10${}^{-19}$	4.3476 × 10${}^{-18}$	6.855 × 10${}^{-19}$	−0.022517	−0.043166
$C_{n_{\beta }}$	$C_{Y_{p}}$	$C_{l_{p}}$	$C_{n_{p}}$	$C_{Y_{r}}$
−0.0608	0.1144	−0.13669	0.0293	−0.14037
$C_{l_{r}}$	$C_{n_{r}}$	$C_{Y_{\delta_{a}}}$	$C_{l_{\delta_{a}}}$	$C_{n_{\delta_{a}}}$
0.026176	−0.038383	−2.8334 × 10${}^{-14}$	1.8034 × 10${}^{-1}$	−9.3649 × 10${}^{-15}$

**Table 4:** Initial parameters during simulation tests with using straight line path.

Test Number	Leader Initial Positions ($L_{pos}$)			Follower Initial Positions ($F_{pos}$)			Desired Distances during Formation Flight		Initial Headings	
	$x_{L}$	$y_{L}$	$z_{L}$	$x_{F}$	$y_{F}$	$z_{F}$	$x_{d}$	$y_{d}$	$\psi_{L}$	$\psi_{F}$
	**[m]**	**[m]**	**[m]**	**[m]**	**[m]**	**[m]**	**[m]**	**[m]**	**[rad]**	**[rad]**
1	100	0	100	0	0	100	−30	−15	0	0
2	100	0	100	0	200	100	−30	−15	0	0
3	100	0	100	0	−200	100	−30	−15	0	0
4	0	0	100	100	0	100	−30	−15	0	π

**Table 5:** Initial parameters during simulation tests with using circle path.

Test Number	Leader Initial Positions ($L_{pos}$)			Follower Initial Positions ($F_{pos}$)			Desired Distances during Formation Flight			Initial Headings	
	$x_{L}$	$y_{L}$	$z_{L}$	$x_{F}$	$y_{F}$	$z_{F}$	$x_{d}$	$y_{d}$	$z_{d}$	$\psi_{L}$	$\psi_{F}$
	**[m]**	**[m]**	**[m]**	**[m]**	**[m]**	**[m]**	**[m]**	**[m]**	**[m]**	**[rad]**	**[rad]**
5	100	0	100	0	0	100	−30	−15	0	0	0
6	0	0	100	100	0	100	−30	−15	0	0	0
7	0	0	100	100	0	100	−30	−15	0	0	π
8	100	0	100	0	0	100	−50	0	−10	0	0

**Table 6:** Statistical parameters of the simulation results.

Test Number	RMSE [m]	RRMSE [%]
1	0.2238	0.6672
2	0.2339	0.6974
3	0.2376	0.7084
4	0.2289	0.6824
5	0.2790	0.8318
6	0.2641	0.7874
7	0.2800	0.8348

**Table 7:** Statistical parameters of the simulation results with communication delay.

Communication Time Delay [sec]	RMSE [m]	RRMSE [%]
0.1	1.1602	3.4590
0.5	1.2438	3.7083
1.0	1.3407	3.9972
1.5	1.4736	4.3934
2.0	1.5844	4.7238

## Data Availability

Not applicable.
